# Pregnancy outcomes and recurrence following surgical treatment of cesarean scar pregnancy: a retrospective analysis

**DOI:** 10.3389/fmed.2025.1650262

**Published:** 2025-09-04

**Authors:** Ruyue Ma, Yu Zheng, Lei Zhang, Ruiheng Zhao, Jian Chen

**Affiliations:** Department of Obstetrics and Gynecology, Suzhou Ninth People’s Hospital, Suzhou Ninth People’s Hospital Affiliated to Soochow University, Suzhou Bay Clinical College of Xuzhou Medical University, Suzhou, China

**Keywords:** cesarean scar pregnancy, hysteroscopy, laparoscopy, reproductive outcomes, intrauterine adhesions

## Abstract

**Background:**

Cesarean scar pregnancy (CSP) is a high-risk ectopic pregnancy, and the influence of surgical treatments on subsequent fertility and pregnancy outcomes remains poorly understood. This study aimed to investigate the impact of different surgical modalities on these outcomes.

**Methods:**

A retrospective analysis was conducted on 460 CSP patients admitted to the Ninth People’s Hospital of Suzhou during the first trimester from January 2015 to December 2023. CSP was categorized into three types based on the implantation site, gestational sac morphology, and the myometrial thickness between the gestational sac and bladder. All patients underwent surgical treatment, which included ultrasound-guided dilation and curettage, hysteroscopic surgery, and combined hysteroscopic and laparoscopic surgery. Clinical information of the patients was systematically collected upon admission, and follow-up data regarding subsequent fertility and pregnancy outcomes were obtained via telephone and verified by medical records. The study outcomes included the incidence of CSP recurrence, secondary infertility, and pregnancy outcomes.

**Results:**

Among the 460 eligible CSP patients, 20 were lost to follow-up. Of the remaining 440 patients, 74 attempted pregnancy after CSP treatment (16.8%). Among these 74 patients, 50 achieved live births (67.6%), 12 developed secondary infertility (16.2%), 2 had an ectopic pregnancy (2.7%), 2 experienced a miscarriage (2.7%), and 8 had CSP recurrence (10.8%). The mean interval between previous CSP treatment and subsequent conception was 16.3 ± 10.83 months. The reproductive outcomes following surgical treatment for CSP were not associated with age, gestational age, number of deliveries, miscarriages, cesarean sections, hospital stay, amenorrhea duration at the time of treatment, maximum diameter of the gestational sac, myometrial thickness of the uterine scar, CSP type, surgical method, or use of methotrexate (MTX) during treatment. However, the number of miscarriages was a contributing factor to secondary infertility, and the presence of post-treatment uterine adhesions was the primary risk factor for failure to achieve pregnancy after CSP surgery.

**Conclusion:**

In the long-term follow-up of women who have undergone CSP treatment, a high success rate in achieving pregnancy and a low recurrence rate were observed. Miscarriages and post-treatment uterine adhesions are risk factors for failure to achieve pregnancy after CSP surgery.

## Introduction

Cesarean scar pregnancy (CSP) refers to a form of ectopic pregnancy in which the fertilized ovum implants at the site of a previous cesarean section in the uterus (1). With the increase in cesarean section rates and advancements in ultrasound technology, the detection rate of CSP has significantly increased (2, 3). Treatment modalities include both surgical and non-surgical methods. Surgical treatments encompass uterine curettage, hysteroscopic surgery, abdominal surgery, or laparoscopic resection (4–7). Non-surgical treatments include systemic administration of methotrexate, local injection of potassium chloride and methotrexate into the gestational sac, needle aspiration, high-intensity focused ultrasound, Foley or Cook catheter insertion, and uterine artery embolization (8, 9). Surgical treatment is the primary approach, with different treatment plans adopted mainly based on the classification of CSP (10, 11).

The current application of diverse treatment strategies, combined with timely diagnosis, has resulted in a high success rate in managing CSP in the majority of cases. The majority of women retain their uterus and fertility after treatment, with a subset expressing a desire to conceive again. However, a significant proportion of CSP patients express no desire for future pregnancies post-surgery, primarily due to concerns regarding the risk of CSP recurrence (12, 13). Studies have indicated that a history of multiple miscarriages, multiple cesarean sections, limited medical resources, and the gestational age and treatment methods used in previous CSP treatments are potential risk factors for recurrent cesarean scar pregnancy (RCSP) (8, 14). Theoretically, since the occurrence of CSP involves blastocyst implantation within the cleft/niche of a previous cesarean scar, excision and repair of the cesarean scar should reduce the recurrence of CSP. There is ongoing debate regarding the use of laparoscopic scar defect repair for improving fertility and clinical pregnancy rates and reducing the risk of RCSP (4, 15). The main adverse pregnancy outcomes reported after CSP include RCSP, ectopic pregnancy, miscarriage, preterm birth, and uterine rupture (8, 13). Despite extensive research, there remains a lack of consensus regarding the influence of various therapeutic modalities on subsequent pregnancy outcomes in women with a history of CSP.

To further investigate the impact of different surgical approaches on reproductive outcomes in CSP patients, we conducted a retrospective cohort study comprising 460 CSP patients who received surgical treatment. The primary objectives of this study were to assess the fertility intentions and outcomes of the patients and to identify potential determinants that may influence fertility outcomes following treatment.

## Materials and methods

### Patients

We retrospectively collected data on patients diagnosed with CSP who underwent surgical treatment at Suzhou Ninth People’s Hospital between January 2015 and December 2023. All cases were first-visit cases with complete medical records and were followed up by telephone for at least 1 year, with follow-up completed in early 2025. A total of 460 patients were initially included in this study. Among them, 20 patients were lost to follow-up due to communication interruption or address change. After excluding patients who did not wish to have children and those lost to follow-up, 74 patients who still desired to conceive again after initial treatment were included in the study, and their clinical data were collected.

The inclusion criteria for the participants were as follows: (1) having a history of at least one previous cesarean section; (2) postoperative pathology indicating normal pregnancy with chorionic villi; (3) meeting the diagnostic criteria for CSP: early pregnancy (≤12 weeks); (4) excluding severe systemic diseases; (5) no emergent massive hemorrhage before surgery; and (6) complete clinical data.

The exclusion criteria were as follows: (1) gestational age > 12 weeks; (2) cesarean scar pregnancy complicated with ectopic pregnancy; (3) concurrent hysterectomy or sterilization, resulting in loss of natural conception ability; and (4) cases referred after ineffective treatment in other hospitals.

CSP patients who met our inclusion criteria were treated as follows: Group A: ultrasound-guided dilation and curettage (D&C); Group B: hysteroscopic removal of uterine scar pregnancy alone or combined with laparoscopic surveillance; and Group C: hysteroscopic removal of cesarean scar pregnancy and laparoscopic uterine repair.

### Data collection

Clinical data of patients were collected through the electronic medical record system of Suzhou Ninth People’s Hospital when they were admitted for CSP treatment, including information such as age, previous pregnancy history, time since the last cesarean section, gestational age, serum β-human chorionic gonadotropin (β-HCG) level before treatment, maximum diameter of gestational sac, and treatment method. Follow-up information related to subsequent fertility and pregnancy outcomes was collected through telephone follow-up. This study was approved by the Ethics Committee of Suzhou Ninth People’s Hospital (registration number: KY-2022-066-01).

### Study design and reproductive outcomes

**Figure 1:**
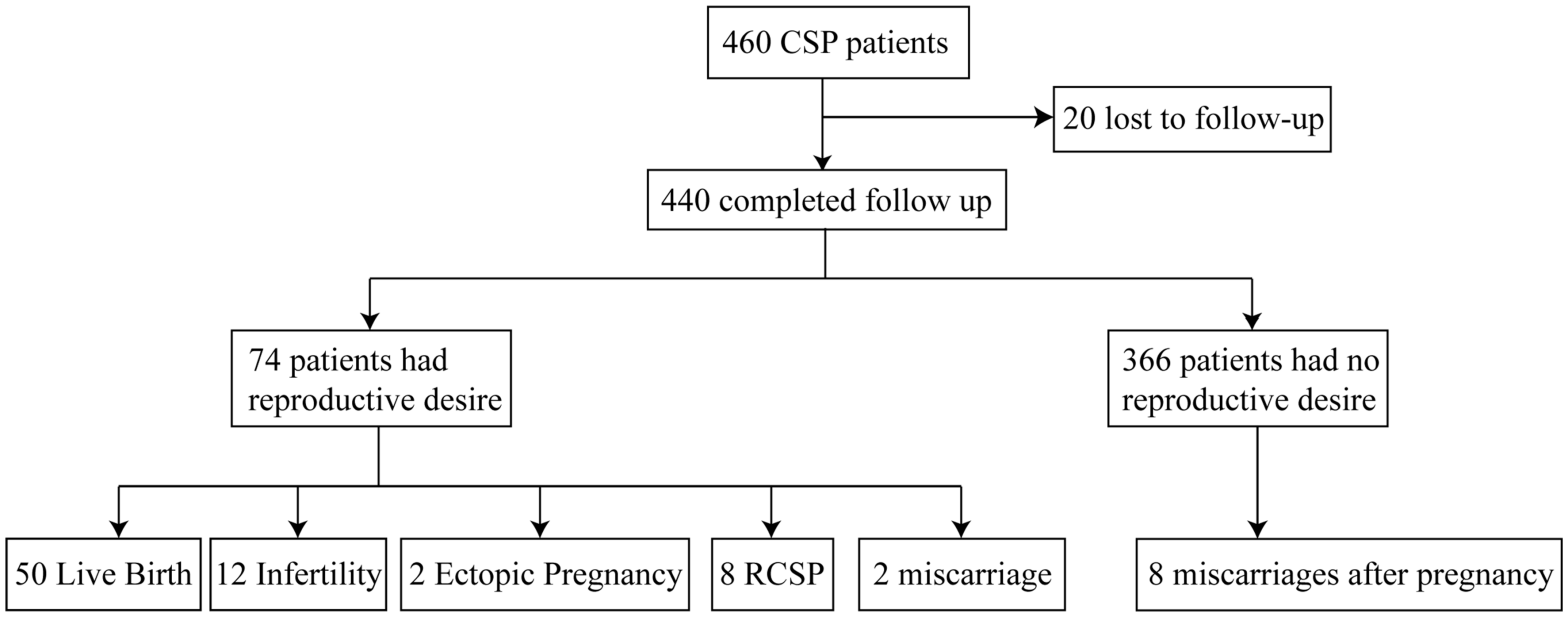
Outcomes of subsequent pregnancies. CSP, cesarean scar pregnancy; RCSP, recurrent cesarean scar pregnancy.

The final reproductive outcomes of the 74 patients included live birth, miscarriage, ectopic pregnancy, recurrent CSP (RCSP), and secondary infertility. Secondary infertility was defined as failure to conceive after 12 months of unprotected sexual intercourse (16). Patients were divided into the live birth group and the non-live birth group based on whether they ultimately achieved a live birth; into the miscarriage group (including spontaneous abortion and miscarriage) and the non-miscarriage group based on whether they had a miscarriage; into the ectopic pregnancy group and the non-ectopic pregnancy group based on whether they had an ectopic pregnancy; and into the RCSP group and the non-RCSP group based on whether CSP recurred. The interval between the end of initial treatment and the telephone follow-up was approximately 1–5 years. Patients who never became pregnant during this period were included in the secondary infertility group, and the remaining patients were included in the non-secondary infertility group. The flowchart of this study is shown in Figure 1.

### CSP types and treatment selection

During the study period, all patients were classified into three types according to the CSP clinical classification system of Qilu Hospital of Shandong University (10). This classification method is based on the location and shape of the gestational sac, blood flow characteristics, and, most importantly, the thickness of the myometrium of the anterior wall scar, which has been described in detail in previous studies (17, 18). Specifically, Type I: Part of the pregnancy tissue is implanted in the uterine scar, and part or most of it is located in the uterine cavity. The myometrium between the pregnancy tissue and the bladder becomes thinner, with a thickness of greater than 3 mm. Type II: Part of the pregnancy tissue is implanted in the uterine scar, and part or most of it is located in the uterine cavity, with some reaching the fundus of the uterine cavity. The myometrium between the pregnancy tissue and the bladder becomes thinner, with a thickness of less than or equal to 0.3 cm but greater than 0.1 cm. Type III: The pregnancy tissue is completely implanted in the muscle layer of the uterine scar and protrudes outward toward the bladder. The uterine cavity and cervical canal are empty. The myometrium between the pregnancy tissue and the bladder is thin or absent, with a thickness of less than or equal to 0.1 cm.

### Surgical methods

The surgical methods used were ultrasound-guided uterine aspiration, hysteroscopic surgery, or laparoscopic pregnancy removal and scar repair. According to the Expert Opinions on the Diagnosis and Treatment of CSP, the choice of surgical treatment depends on patient preferences, surgical indications and contraindications, and the professional knowledge and advice of the clinical physician. Details of the specific surgical methods can be found in our previous study (19). During the D&C procedure, the entire operation is carried out under strict ultrasonic monitoring. Gentle suction is used to carefully remove the ectopic tissue from the cesarean scar. The operation continues until the appearance of villous tissue in the aspirate and the ultrasound shows a clear endometrial line with no residual gestational sac, which indicates that the pregnancy tissue in the uterus has been completely cleared.

Hysteroscopic surgery or combined laparoscopic lesion resection is performed by experienced gynecologists. The patient will receive intravenous anesthesia or general anesthesia. Under the guidance of the hysteroscope, the ectopic pregnancy tissue and its surrounding blood flow can be directly observed. First, the majority of the tissue is clamped with oval forceps and, then, negative-pressure uterine aspiration is performed. Finally, a comprehensive inspection of the uterine cavity is performed using a hysteroscope to confirm the absence of residual tissue.

When performing laparoscopic repair of a cesarean scar pregnancy, the bladder peritoneum is first opened to expose the vesicouterine space, which is convenient for exposing the uterine isthmus. During the operation, a hysteroscope with a light source is used to accurately locate the CSP lesion. Then, an ultrasonic scalpel is used to completely excise the scar area, including the pregnancy tissue. After the excision, the defective part of the uterus is thoroughly irrigated, trimmed, and sutured. The tissue resected during the operation will be sent for pathological examination.

### Statistical analysis

The data were processed using the Statistical Package for the Social Sciences (SPSS) 26.0 statistical software. Continuous variables were expressed as mean ± standard deviation. For the comparison between two groups, a two-sample independent *t*-test was employed. Categorical data were presented in the form of frequencies and percentages and were analyzed using either the chi-squared test or Fisher’s exact probability method. A *p*-value of less than 0.05 was considered statistically significant.

## Results

### Pregnancy outcomes after CSP treatment

Among the 460 women who met the inclusion criteria for cesarean scar pregnancy (CSP), 20 were lost to follow-up. Of the remaining 440 women, 74 attempted to conceive after CSP treatment (74/440, 16.81%). Among these 74 women, 50 (50/74, 67.57%) achieved live birth, 12 (12/74, 16.22%) developed secondary infertility after CSP treatment, 2 (2/74, 2.70%) experienced an ectopic pregnancy, 2 (2/74, 2.70%) had a miscarriage, and 8 (8/74, 10.81%) had recurrent CSP. Among the 50 women with live births, 2 (2/74, 2.70%) underwent preterm cesarean delivery due to fetal distress, and 2 had placenta previa (2/74, 2.70%). In the non-live birth group, intrauterine adhesions (IUAs) occurred in 10 women (10/24, 41.67%).

### Analysis of factors affecting live birth outcomes in CSP patients

A univariate analysis revealed that the incidence of uterine adhesions was significantly lower in the live birth group than in the non-live birth group (*p* < 0.002, Table 1). However, there were no statistically significant differences between the two groups in terms of patient age, duration of amenorrhea during treatment, number of cesarean sections, number of miscarriages, time since last cesarean section, mean gestational sac diameter, maximum gestational sac diameter, myometrial thickness of the uterine scar, CSP type, treatment method, preoperative serum HCG levels, intraoperative blood loss, and hospital stay (*p* > 0.05, Table 1).

**Table 1:** Comparison of clinical data between the live birth group and the non-live birth group after treatment for CSP.

Clinical characteristics	Live birth group (*n* = 50)	Non-live birth group (*n* = 24)	Statistic	*p*-value
Age (years)	30.24 ± 3.48	30.50 ± 4.70	0.190	0.851
Gestational age (days)	49.64 ± 46.48	31.00 ± 23.18	−1.307	0.200
Number of cesarean deliveries	1.64 ± 1.58	1.33 ± 0.65	−0.644	0.524
Number of miscarriages	1.20 ± 0.41	1.33 ± 0.50	0.870	0.390
Interval between the previous CS and the present (years)	6.72 ± 2.24	6.64 ± 2.42	−0.097	0.923
Mean diameter of the gestational sac (mm)	26.46 ± 19.34	18.72 ± 3.71	−1.928	0.064
Maximum diameter of the gestational sac (mm)	33.08 ± 23.74	26.67 ± 5.77	−1.274	0.213
CSP type			1.361	0.559
Type I	32 (64%)	16 (66.67%)		
Type II	12 (24%)	8 (33.33%)		
Type III	6 (12%)	0 (0%)		
Uterine synechiae			13.006	<0.002
Yes	0 (0%)	10 (41.67%)		
No	50 (100%)	14 (58.33%)		
Thickness of myometrium (mm)	3.93 ± 2.41	4.54 ± 1.81	0.766	0.449
Blood HCG value (U/L)	47344.10 ± 47429.15	43042.67 ± 49133.40	−0.255	0.800
Surgical method			1.095	0.787
A	2 (4%)	0 (0%)		
B	34 (68%)	20 (83.33%)		
C	14 (28%)	4 (16.67%)		
Operative time (minutes)	49.64 ± 46.48	31.00 ± 23.18	−1.307	0.200
Intraoperative bleeding (ml)	34.00 ± 36.37	56.25 ± 109.11	0.929	0.359
MTX			0.009	1.000
Yes	20 (40.00%)	10 (41.67%)		
No	30 (60.00%)	14 (58.33%)		
Duration of hospitalization (days)	6.32 ± 2.64	4.83 ± 0.93	−1.882	0.068
Expenses (¥)	9570.61 ± 9680.03	6141.53 ± 2507.54	−1.200	0.238

Measurement data were expressed as (mean ± SD); enumeration data were expressed as *n* (%).

CS, cesarean section; CSP, cesarean scar pregnancy; MTX, methotrexate.

### Analysis of factors affecting the recurrence of CSP in patients

The univariate analysis showed no statistically significant differences between the non-RCSP group and the RCSP group in terms of patient age, duration of amenorrhea during treatment, number of cesarean sections, number of miscarriages, time since the last cesarean section, mean gestational sac diameter, maximum gestational sac diameter, myometrial thickness of the uterine scar, CSP type, treatment method, preoperative serum HCG levels, intraoperative blood loss, hospital stay, and presence of uterine adhesions (*p* > 0.05, Table 2). The results indicated that laparoscopic repair of scar defects did not reduce the risk of RSCP. It is noteworthy that all eight recurrent cases in Group B had undergone surgery, comprising four type I and four type II cases, with no type III recurrences. The mean time to recurrence was 17.5 ± 5.45 months; all cases were detected early, and none had intrauterine adhesions after the initial procedure.

**Table 2:** Comparison of clinical data between the RCSP and non-RCSP groups after treatment for CSP.

Clinical characteristics	Non-RCSP group (*n* = 66)	RCSP group (*n* = 8)	Statistic	*p*-value
Age (years)	30.21 ± 3.97	31.25 ± 2.99	−0.504	0.618
Gestational age (days)	46.61 ± 42.42	18.75 ± 8.54	0.115	0.204
Number of cesarean deliveries	1.52 ± 1.42	1.75 ± 0.50	−2.669	0.011
Number of miscarriages	1.18 ± 0.39	1.75 ± 0.50	−0.326	0.747
Interval between the previous CS and the present (years)	6.88 ± 2.30	5.00 ± 1.00	1.384	0.178
Mean diameter of the gestational sac (mm)	25.38 ± 16.86	13.33 ± 2.23	1.109	0.281
Maximum diameter of the gestational sac (mm)	32.54 ± 20.43	18.25 ± 6.95	1.394	0.172
CSP type			1.376	0.694
Type I	44 (66.67%)	4 (50.00%)		
Type II	16 (24.24%)	4 (50.00%)		
Type III	6 (9.10%)	0 (0%)		
Uterine synechiae			1.235	0.624
Yes	10 (15.15%)	0 (0%)		
No	56 (84.85%)	8 (100%)		
Thickness of myometrium (mm)	4.27 ± 2.29	2.98 ± 1.13	1.094	0.281
Blood HCG value (U/L)	45762.65 ± 49816.43	47486.75 ± 20569.71	0.218	0.946
Surgical method			1.771	0.601
A	2 (3.03%)	0 (0%)		
B	46 (69.70%)	8 (100%)		
C	18 (27.27%)	0 (0%)		
Operative time (minutes)	46.61 ± 42.42	18.75 ± 8.54	1.295	0.204
Intraoperative bleeding (ml)	44.55 ± 71.36	13.75 ± 11.09	0.851	0.400
MTX			0.164	0.686
Yes	26 (39.39%)	4 (50.00%)		
No	40 (60.61)	4 (50.00%)		
Duration of hospitalization (days)	6.00 ± 2.41	4.50 ± 0.57	1.226	0.228
Expenses (¥)	8912.10 ± 8569.24	4716.15 ± 497.59	0.967	0.340

Measurement data were expressed as (mean ± SD); enumeration data were expressed as *n* (%).

CS, cesarean section; CSP, cesarean scar pregnancy; MTX, methotrexate.

### Analysis of factors affecting secondary infertility outcomes in CSP patients

We compared the clinical characteristics of patients with secondary infertility and found that, compared to the non-secondary infertility group, patients in the secondary infertility group had a higher incidence of postoperative uterine adhesions and a higher number of previous miscarriages, while other indicators showed no statistically significant differences (*p* > 0.05, Table 3).

**Table 3:** Comparison of clinical data between the infertility and non-infertility groups after treatment for CSP.

Clinical characteristics	Non-infertility group (*n* = 62)	Infertility group (*n* = 12)	Statistic	*p*-value
Age (years)	30.58 ± 3.70	29.00 ± 4.69	0.918	0.365
Gestational age (days)	45.68 ± 43.59	32.83 ± 23.46	0.697	0.490
Number of cesarean delivery	1.61 ± 1.45	1.17 ± 0.48	0.739	0.465
Number of miscarriages	1.29 ± 0.46	1.00 ± 0.00	3.503	0.001
Interval between the previous CS and the present (years)	6.83 ± 2.42	6.17 ± 1.60	0.627	0.536
Mean diameter of the gestational sac (mm)	24.83 ± 17.70	19.36 ± 3.79	0.747	0.460
Maximum diameter of the gestational sac (mm)	31.93 ± 21.51	26.17 ± 5.91	0.645	0.523
CSP type			0.746	0.794
Type I	38 (61.29%)	10 (83.33%)		
Type II	18 (29.03%)	2 (16.67%)		
Type III	6 (9.67%)	0 (0%)		
Uterine synechiae			29.870	<0.001
Yes	0 (0%)	10 (83.33%)		
No	62 (100%)	2 (16.67%)		
Thickness of myometrium (mm)	3.92 ± 2.28	5.27 ± 1.67	−1.377	0.177
Blood HCG value (U/L)	47268.01 ± 48833.85	39134.33 ± 41996.88	0.381	0.706
Surgical method			0.760	1.000
A	2 (3.23%)	0 (0%)		
B	44 (70.97%)	10 (83.33%)		
C	16 (25.81%)	2 (16.67%)		
Operative time (minutes)	45.68 ± 43.59	32.83 ± 23.46	0.697	0.490
Intraoperative bleeding (ml)	43.39 ± 74.08	30.00 ± 15.49	0.436	0.665
MTX			0.154	1.000
Yes	36	8		
No	26	4		
Duration of hospitalization (days)	6.00 ± 2.47	5.00 ± 1.09	0.962	0.342
Expenses (¥)	8990.46 ± 8851.31	5761.56 ± 1477.88	0.879	0.386

Measurement data were expressed as (mean ± SD); enumeration data were expressed as *n* (%).

CS, cesarean section; CSP, cesarean scar pregnancy; MTX, methotrexate.

### Analysis of reproductive outcomes across different CSP types and surgical approaches

Our findings indicate that patients with type III achieved the best outcomes (100% live birth rate, no recurrence) when treated with combined hysteroscopic–laparoscopic surgery, whereas type II patients had a higher recurrence rate (20.0%). Group A exhibited a 100% live birth rate, with no cases of secondary infertility or recurrent CSP; Group B, the most widely used protocol, was associated with the highest recurrence rate; and Group C had no recurrent CSP cases (Table 4).

**Table 4:** Stratified analysis of reproductive outcomes by CSP type and surgical method.

Clinical characteristics	RCSP rate	Live birth rate	Infertility rate
CSP type			
Type I	4 (8.33%)	32 (66.67%)	10 (20.83%)
Type II	4 (20%)	12 (60%)	2 (10%)
Type III	0 (0%)	6 (100%)	0 (0%)
Surgical method			
A	0 (0%)	2 (100%)	0 (0%)
B	8 (14.81%)	34 (62.96%)	10 (18.52%)
C	0 (0%)	14 (77.78%)	2 (11.11%)

CSP, cesarean scar pregnancy.

## Discussion

The relationship between CSP treatment and subsequent fertility and pregnancy outcomes has not been sufficiently investigated. The majority of women do not plan to conceive after CSP treatment, primarily due to concerns about pregnancy-related risks and the recurrence of CSP (12, 20). Therefore, we conducted a retrospective study, including as many samples as possible with a follow-up period exceeding 2 years, to determine the factors affecting pregnancy outcomes after CSP treatment. The results showed that CSP patients had a high rate of subsequent pregnancy and a low risk of RCSP, while the number of previous miscarriages and post-treatment uterine adhesions were the main risk factors for failure to achieve pregnancy after CSP surgery.

In 2022, the Society for Maternal-Fetal Medicine (SMFM) stated that patients with CSP may attempt pregnancy after uterine-sparing therapy but should be counseled about the substantial risks of recurrence and severe maternal morbidity. Published data indicate a CSP recurrence rate of 5–40%, and subsequent pregnancies are associated with placenta accreta spectrum, spontaneous uterine rupture, stillbirth, and other adverse outcomes (13, 21). In the present study, the recurrence rate was 10.81% (8/74), which aligns with the findings reported by Ben Nagi et al. (22). The observed variability in recurrence rates across studies largely reflects differences in sample size, patient selection, and follow-up duration (8, 16, 23). By strictly stratifying CSP types and matching them to corresponding surgical approaches, our study provides clinicians with more tailored guidance. Notably, all eight recurrent cases had undergone either hysteroscopic resection of the CSP lesion alone or hysteroscopic resection under laparoscopic surveillance. It remains unclear whether failure to repair the uterine scar defect is associated with a higher risk of recurrence (16, 24–26); nonetheless, these findings underscore the potential importance of scar repair. Laparoscopic repair allows direct suturing of the scar defect, thereby reducing anatomic distortion of the isthmus—an essential prerequisite for blastocyst implantation within the scar (19, 27). Prospective studies are warranted to determine whether routine scar repair should be recommended for patients desiring future pregnancy.

In our study, neither the type of surgery nor the classification of CSP affected the subsequent pregnancy outcomes in CSP patients. In a study involving 51 CSP patients planning subsequent pregnancies, 31 (60.8%) achieved full-term pregnancies, 2 experienced complications, 2 had placenta accreta, and 1 required a hysterectomy due to hemorrhage during cesarean section (23). In another study involving 166 subsequent pregnancies in CSP patients, 58 (34.94%) resulted in normal pregnancies. Among these, 4 developed gestational diabetes, 2 had hypertensive disorders, 2 experienced placenta previa with the placenta located on the posterior wall, and 1 experienced preterm rupture of membranes in the late stage of pregnancy (28). In this study, fetal distress and placenta previa were also observed among patients who achieved pregnancy. Although women with a history of CSP face risks of recurrent CSP and other severe maternal morbidities in subsequent pregnancies, the probability of retaining reproductive function after CSP treatment and attempting to conceive again is relatively high. Our findings demonstrate that the incidence of intrauterine adhesions was significantly lower in the live-birth group than in the non-live-birth group, independent of CSP type or surgical modality. Therefore, regardless of the surgical procedure, excessive curettage should be avoided intraoperatively.

Currently, research on infertility after treatment in CSP patients is largely based on medical records, with insufficient evidence demonstrating how different treatment plans impact subsequent fertility. Previous studies have reported an overall infertility incidence of 15.7% (8/51) after conservative and surgical treatment for CSP (23). Another study reported that among 79 CSP patients who underwent ultrasound-guided uterine curettage, 13 (16.5%) experienced infertility (29). In this study, the incidence of infertility was 16.22% (12/74), which is consistent with the previous literature. Our data indicate that secondary infertility after CSP surgery is not associated with the surgical method itself but is more likely to occur in patients with a history of multiple abortions. We speculate that repeated curettage procedures may compromise the integrity of the endometrium, impair the myometrium, and increase the risk of iatrogenic endometriosis at the cesarean scar defect area (9, 30), thereby increasing the risk of infertility. Furthermore, both the incidence and severity of intrauterine adhesions are key factors influencing pregnancy outcomes after CSP treatment (31). Recurrent miscarriages—especially those managed by curettage—can injure the basal layer of the endometrium, resulting in reduced endometrial glands and impaired angiogenesis, which in turn compromises endometrial receptivity (32, 33). Concomitantly, repeated pregnancy losses may trigger a localized inflammatory response in the endometrium—manifested by elevated pro-inflammatory cytokines such as TNF-α and IL-6—that inhibits embryo implantation (34). Intrauterine adhesions mechanically obstruct the uterine cavity, impeding embryonic migration, and disrupt endometrial blood perfusion, resulting in local hypoxia (35). Moreover, the fibrotic tissue within adhesion areas decreases estrogen sensitivity and impedes cyclic endometrial proliferation, further weakening the capacity for embryo implantation (36). In this study, intrauterine adhesions were the principal factor contributing to secondary infertility after CSP surgery. This finding underscores the need to adopt the least endometrium-damaging method when pregnancy termination is required in CSP patients and to prioritize postoperative adhesion prevention in those wishing to conceive. Adhesions should be promptly lysed upon detection. For patients with a history of repeated miscarriages, pre-conception assessment of endometrial receptivity is advised, and avoidable curettage should be eschewed to minimize further endometrial injury.

Our study has several inherent limitations. First, as a single-center, retrospective cohort study, it is susceptible to selection bias that may compromise the validity and generalizability of the findings. Second, because a substantial proportion of participants had no desire for subsequent pregnancy after CSP treatment, analyses of reproductive and obstetric outcomes are inevitably underpowered and restricted to a small subgroup. Third, only three surgical strategies were evaluated; patients managed with alternative approaches—such as expectant or medical therapy, transvaginal excision, or high-intensity focused ultrasound—were not included, leaving important therapeutic comparisons unexplored. Fourth, lifestyle-related factors, including dietary habits, physical activity levels, smoking status, alcohol consumption, and psychological stress, were not systematically assessed or incorporated into the analyses. These factors are known to potentially influence both the recovery process after CSP treatment and subsequent reproductive health outcomes, which may introduce confounding effects and limit the comprehensiveness of our findings. For instance, inadequate nutrition or sedentary behavior could impact uterine healing, while chronic stress might affect hormonal balance and fertility potential, all of which could interact with the surgical outcomes we evaluated but were not accounted for in our study design. Consequently, multicenter, prospective, randomized controlled trials with larger sample sizes and extended follow-up are warranted to minimize bias and provide higher-level evidence. Although our results highlight the critical role of preventing intrauterine adhesions in fertility-seeking individuals after CSP, these observations require validation in future well-designed studies that also consider a broader range of confounding variables, including lifestyle and behavioral factors.

## Conclusion

In the long-term follow-up of women treated for CSP, we observed high subsequent fertility and a low rate of RCSP. Reproductive outcomes did not differ significantly among surgical approaches, and the overall prognosis after CSP treatment was favorable. For patients desiring future fertility, proactive prevention of intrauterine adhesions is essential to avert secondary infertility. These findings provide further insight into optimizing CSP management for women who wish to preserve reproductive capacity. However, further studies are still warranted to validate the results of this study.

## Data Availability

The original contributions presented in the study are included in the article/supplementary material, further inquiries can be directed to the corresponding author.
